# Supplementary figures and images for: SDC4 drives fibrotic remodeling of the intervertebral disc under altered spinal loading
Source: Cell Death Dis. 2025 Oct 6;16(1):678. doi: 10.1038/s41419-025-08002-3 (PMC12500954; doi:10.1038/s41419-025-08002-3)

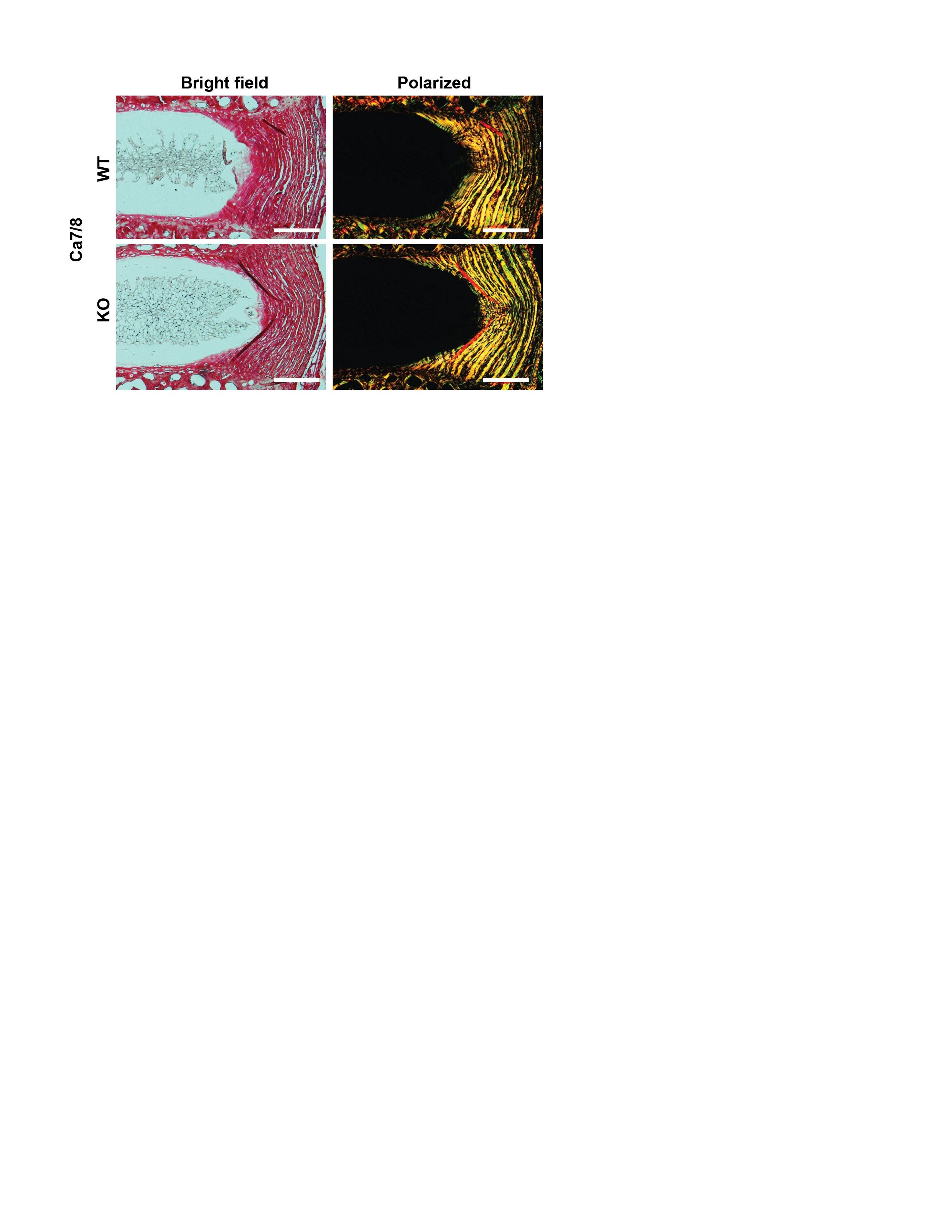

Supplement: Supplementary file 2 — Supplementary Figure S1 [file 41419_2025_8002_MOESM2_ESM.png]

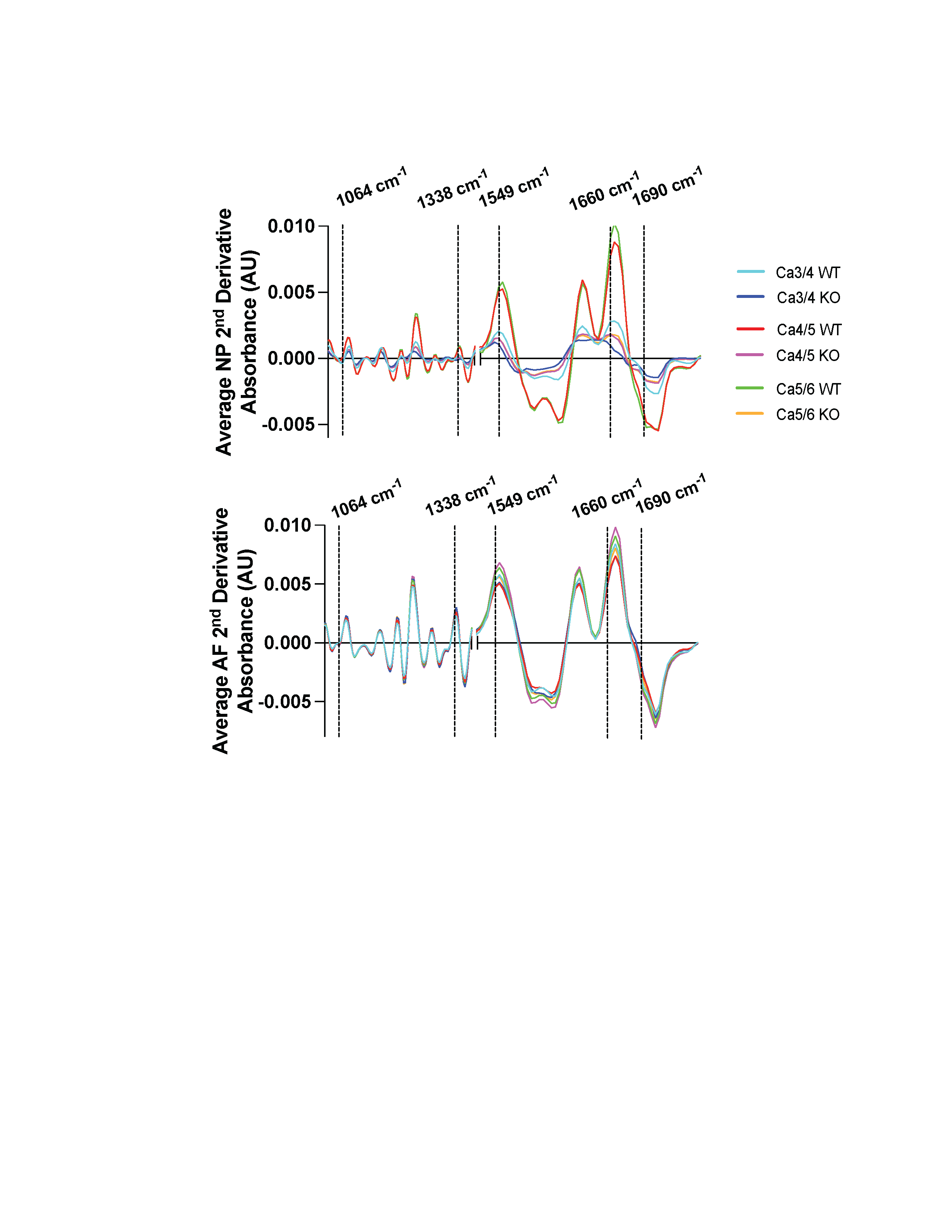

Supplement: Supplementary file 3 — Supplementary Figure S2 [file 41419_2025_8002_MOESM3_ESM.png]

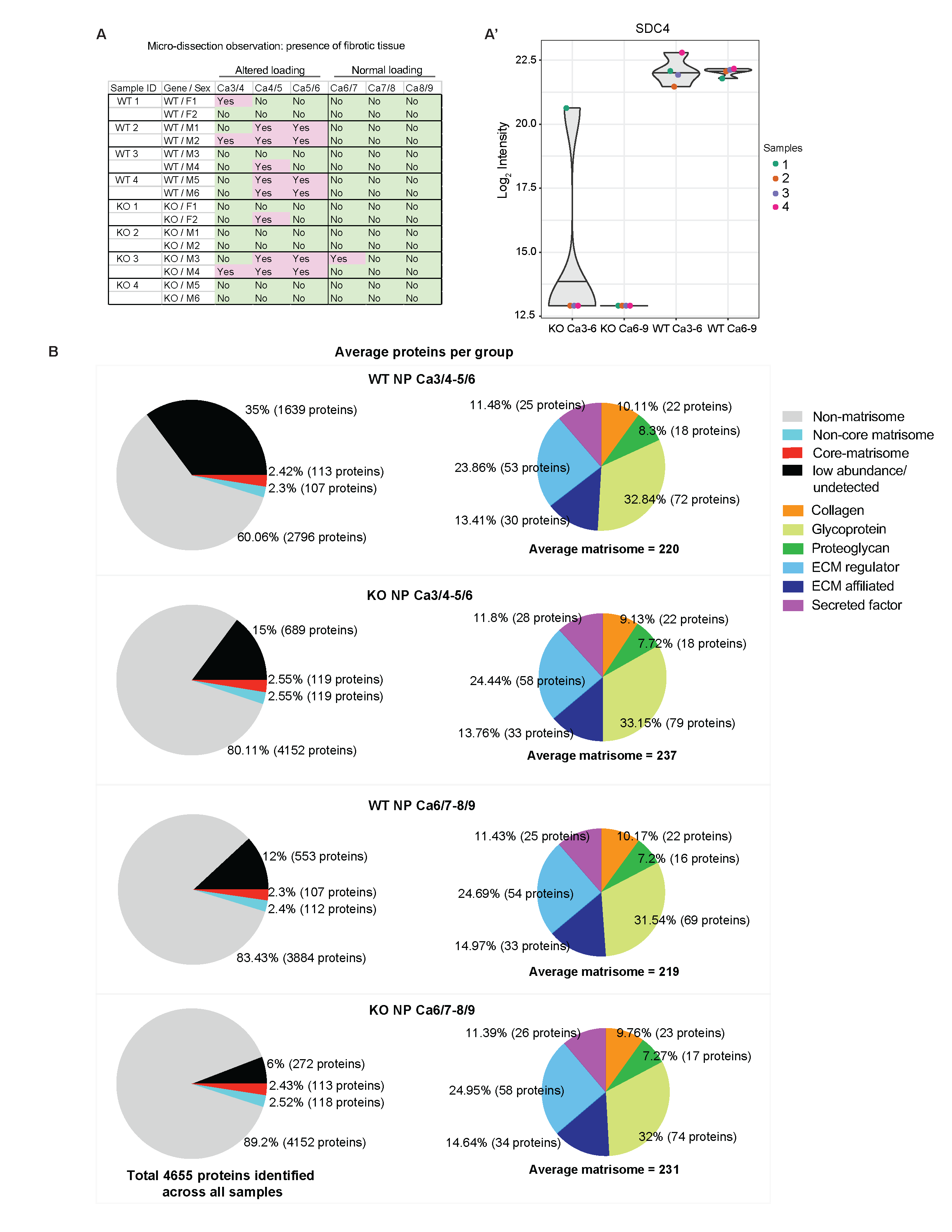

Supplement: Supplementary file 4 — Supplementary Figure S3 [file 41419_2025_8002_MOESM4_ESM.png]

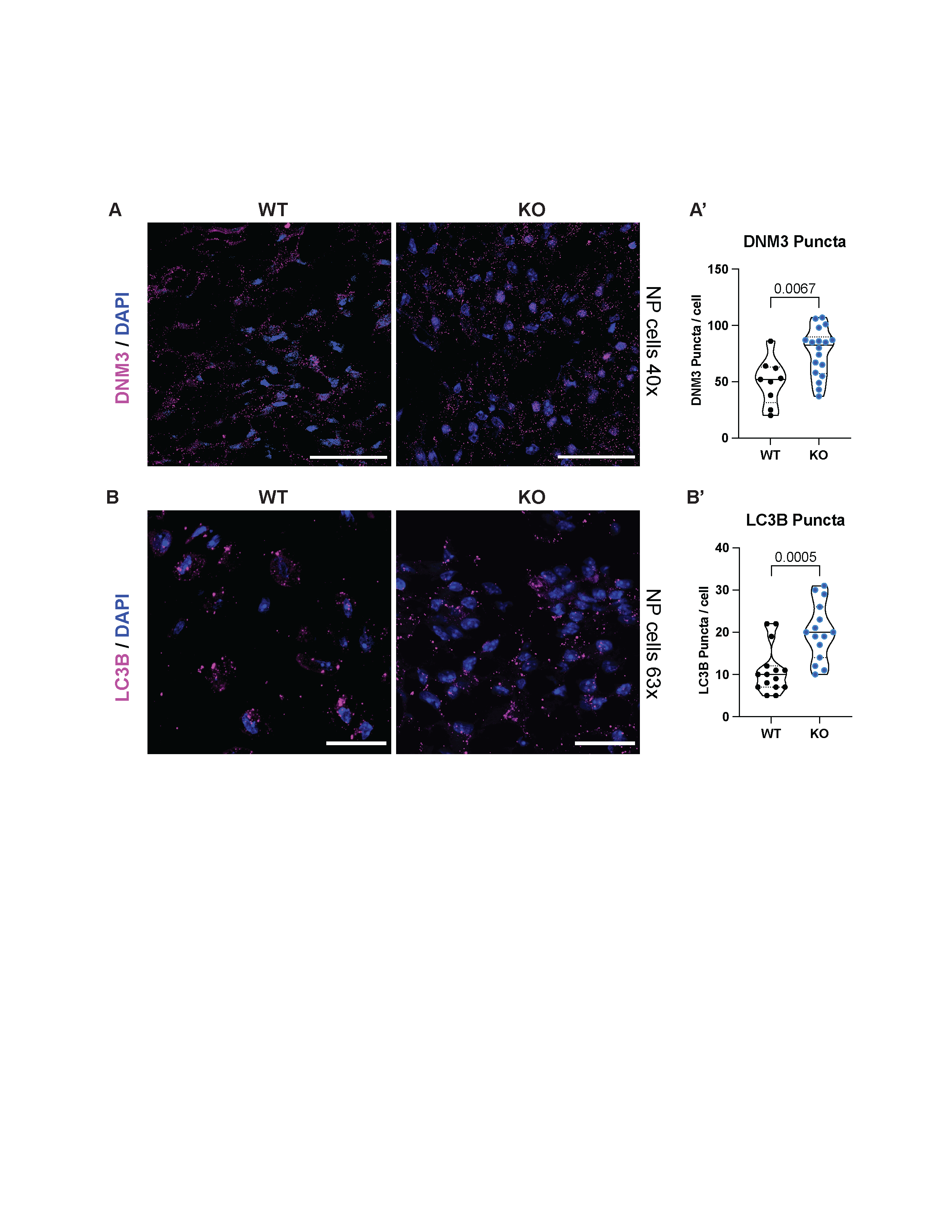

Supplement: Supplementary file 5 — Supplementary Figure S4 [file 41419_2025_8002_MOESM5_ESM.png]

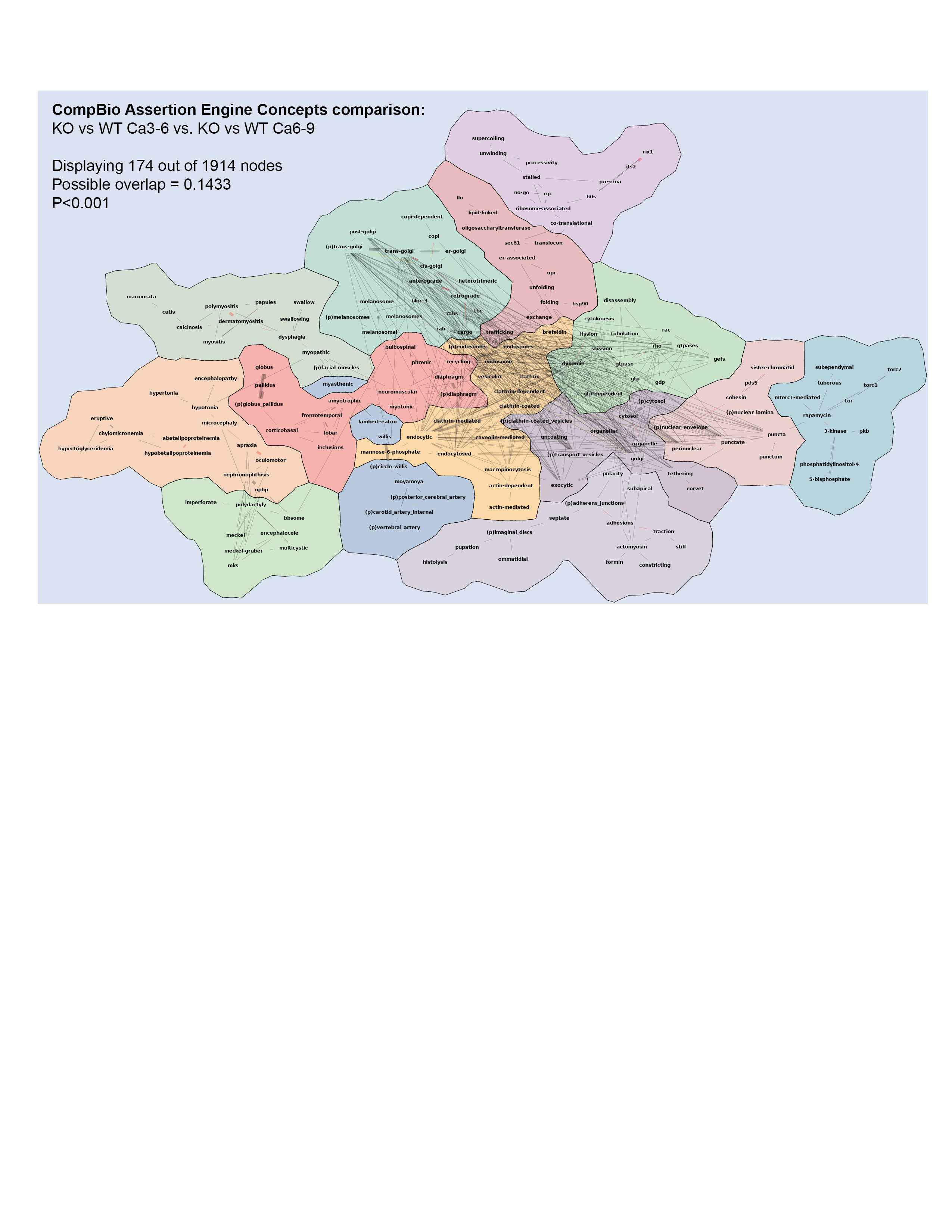

Supplement: Supplementary file 6 — Supplementary Figure S5 [file 41419_2025_8002_MOESM6_ESM.png]

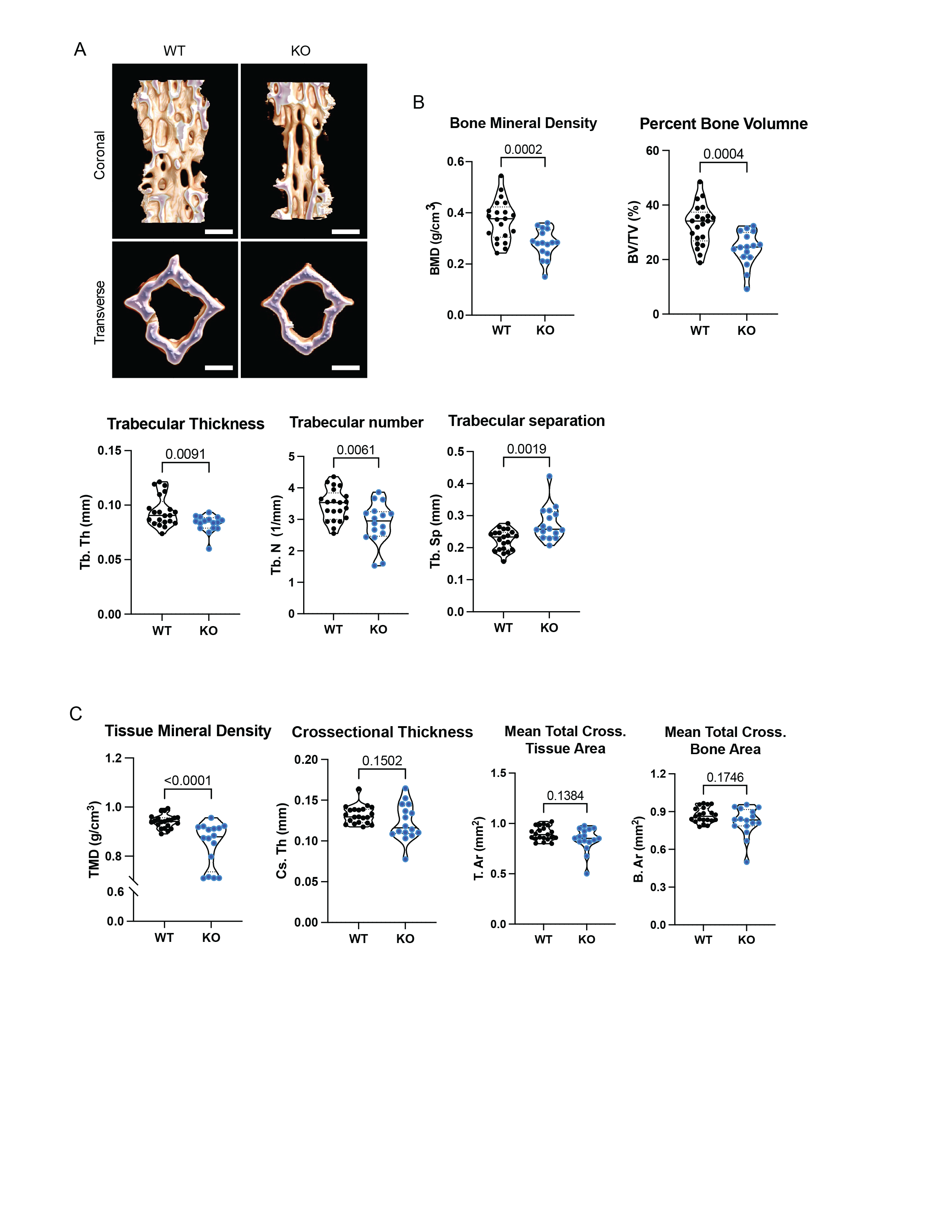

Supplement: Supplementary file 7 — Supplementary Figure S6 [file 41419_2025_8002_MOESM7_ESM.png]
